# Type B insulin resistance syndrome associated with connective tissue disease and psoriasis

**DOI:** 10.1530/EDM-20-0027

**Published:** 2020-08-04

**Authors:** Agnieszka Łebkowska, Anna Krentowska, Agnieszka Adamska, Danuta Lipińska, Beata Piasecka, Otylia Kowal-Bielecka, Maria Górska, Robert K Semple, Irina Kowalska

**Affiliations:** 1 Department of Internal Medicine and Metabolic Diseases, Diabetology and Internal Medicine; 2 Department of Endocrinology, Diabetology and Internal Medicine; 3 Department of Rheumatology and Internal Diseases, Medical University of Bialystok, Bialystok, Poland; 4 Centre for Cardiovascular Science, Queen’s Medical Research Institute, University of Edinburgh, Edinburgh, UK

**Keywords:** Adult, Male, White, Poland, Pancreas, Skin, Diabetes, Insulin, Insulin resistance, Psoriasis*, Connective tissue disorders*, Autoimmune disorders, Hypoglycaemia, Insulin resistance, Hypoglycaemia, Psoriatic arthritis, Psoriasis*, Acanthosis nigricans, Weight loss, Fatigue, Raynaud's syndrome*, Sclerodactyly*, Enlarged parotid glands*, Palpable lymph nodes*, Tachycardia, Anaemia, Thrombocytopenia, Leukopenia*, Lymphopenia*, Glucosuria, Anti-insulin antibodies, Anti-insulin receptor antibodies*, Immunoprecipitation*, Hyperinsulinaemic euglycaemic clamp*, DEXA scan, BMI, White blood cell count, Red blood cell count, Haemoglobin A1c, Insulin, Antinuclear antibody, Coombs test*, Antiribonucleoprotein antibodies*, Adiponectin, Triglycerides, Glucose (urine), Urinalysis, Complement*, Platelet count, Alanine aminotransferase*, Sodium, Metformin, Insulin, Corticosteroids, Hydroxychloroquine*, Methotrexate*, Methylprednisolone, Prednisone, Dermatology, New disease or syndrome: presentations/diagnosis/management, August, 2020

## Abstract

**Summary:**

Type B insulin resistance syndrome (TBIR) is characterised by the rapid onset of severe insulin resistance due to circulating anti-insulin receptor antibodies (AIRAs). Widespread acanthosis nigricans is normally seen, and co-occurrence with other autoimmune diseases is common. We report a 27-year-old Caucasian man with psoriasis and connective tissue disease who presented with unexplained rapid weight loss, severe acanthosis nigricans, and hyperglycaemia punctuated by fasting hypoglycaemia. Severe insulin resistance was confirmed by hyperinsulinaemic euglycaemic clamping, and immunoprecipitation assay demonstrated AIRAs, confirming TBIR. Treatment with corticosteroids, metformin and hydroxychloroquine allowed withdrawal of insulin therapy, with stabilisation of glycaemia and diminished signs of insulin resistance; however, morning fasting hypoglycaemic episodes persisted. Over three years of follow-up, metabolic control remained satisfactory on a regimen of metformin, hydroxychloroquine and methotrexate; however, psoriatic arthritis developed. This case illustrates TBIR as a rare but severe form of acquired insulin resistance and describes an effective multidisciplinary approach to treatment.

**Learning points:**

- We describe an unusual case of type B insulin resistance syndrome (TBIR) in association with mixed connective tissue disease and psoriasis.
- Clinical evidence of severe insulin resistance was corroborated by euglycaemic hyperinsulinaemic clamp, and anti-insulin receptor autoantibodies were confirmed by immunoprecipitation assay.
- Treatment with metformin, hydroxychloroquine and methotrexate ameliorated extreme insulin resistance.

## Background

Type B insulin resistance syndrome (TBIR) is a rare autoimmune disorder characterised by the development of anti-insulin receptor antibodies (AIRA). It most commonly leads to extreme insulin resistance with severe hyperglycaemia and weight loss; however, hypoglycaemia may be seen, especially where antibody titre is low (1). TBIR is commonly associated with underlying autoimmune diseases, such as systemic lupus erythematosus (SLE), various connective tissue diseases, interstitial lung disease or organ-specific dermatological, haematological, or hepatic disorders (1, 2, 3). Here, we present an unusual case of TBIR associated with connective tissue disease and psoriasis, with insulin resistance confirmed by hyperinsulinaemic euglycaemic clamp and stabilised with a regimen of corticosteroids, hydroxychloroquine and metformin.

## Case presentation

A 27-year-old Caucasian man with a 2-year history of psoriasis was hospitalised because of an unexplained weight loss of 20-kg in one year. He also reported fatigue and a new onset of Raynaud’s phenomenon. One year earlier, he had undergone haematological evaluation because of peripheral lymphadenopathy and enlarged parotid glands, with results deemed consistent with viral infection. Lymphoproliferative disease was excluded on the basis of lymph node histopathological examination. There was no family history of diabetes or autoimmune disease.

## Investigation

**Figure 1:**
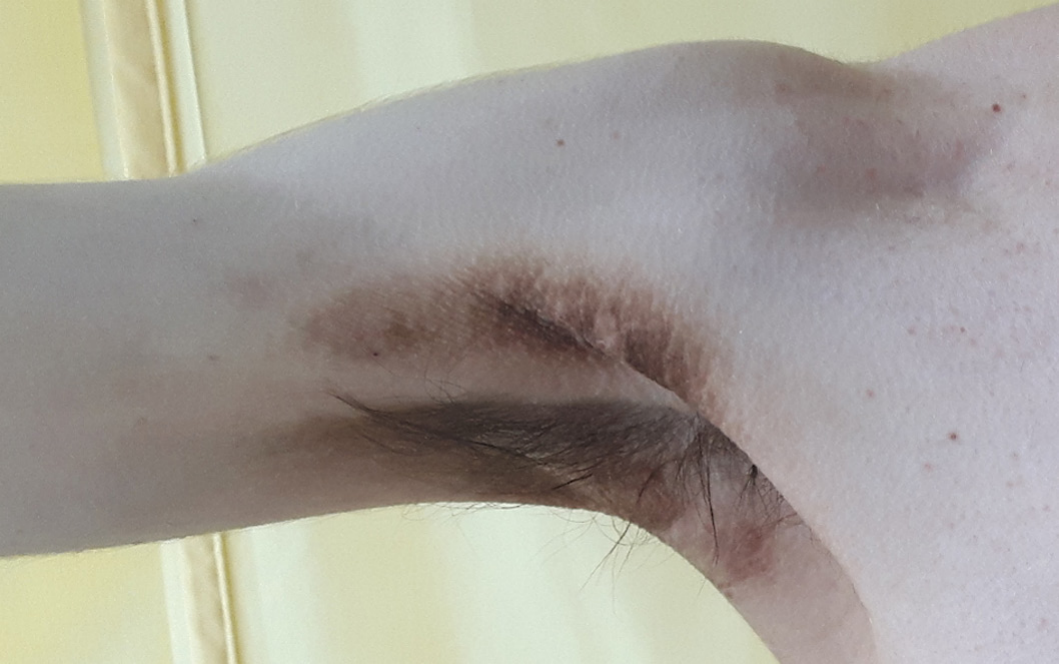
The extent of acanthosis nigricans in the patient on admission.

**Figure 2:**
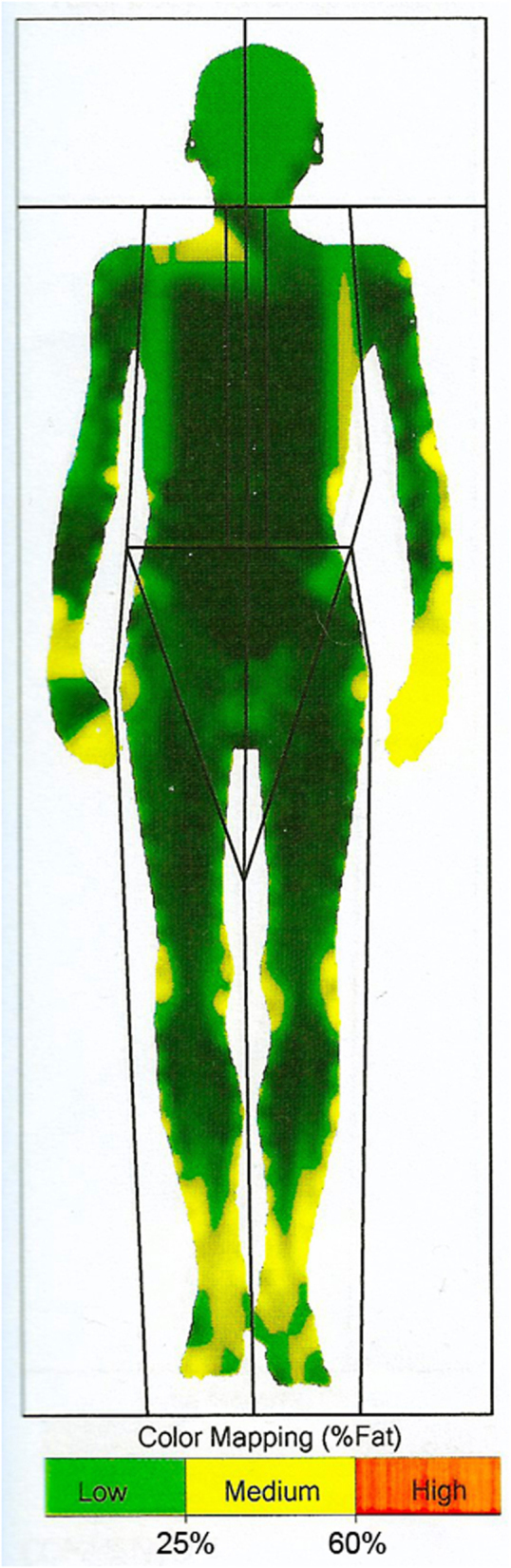
Distribution of adipose tissue obtained by dual energy X-ray absorptiometry.

**Figure 3:**
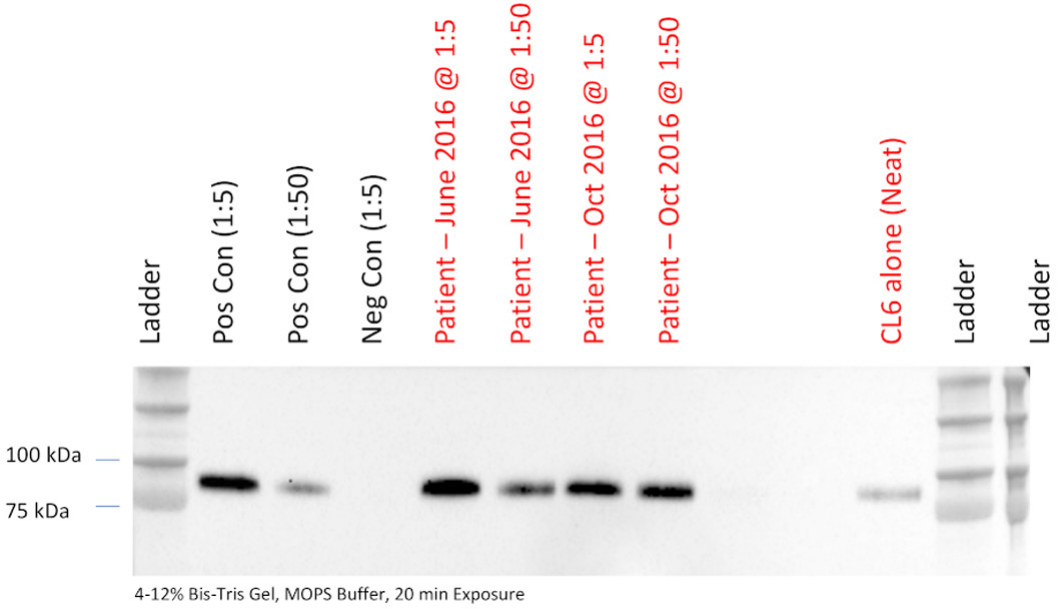
Detection of anti-insulin receptor autoantibodies. Anti-insulin receptor autoantibodies were detected by immunoprecipitation assay using input of serum diluted either 1:5 or 1:50 as indicated. Anti-insulin receptor probing of immunoprecipitated is shown. +ve control, positive control serum obtained from a patient with proven type B insulin resistance with high antibody titre. ‘‘CL6’ denotes lysate from Chinese Hamster Ovary Cells stably expressing human insulin receptor as an additional control. Times denote interval from initial presentation.

Physical examination revealed malnutrition. Height was 180 cm and weight 53.3 kg (BMI 16.4 kg/m^2^). There was widespread acanthosis nigricans in the axillae, antecubital fossae, groins, and on the thumbs (Fig. 1). Sclerodactyly, psoriatic lesions, enlarged parotid glands, palpable cervical and axillary lymph nodes, and tachycardia were also noted. Dual energy X-ray absorptiometry revealed a paucity of subcutaneous and visceral fat and decreased lean body mass (Fig. 2). Table 1 shows laboratory findings on admission. Full blood count revealed anaemia, leucopaenia with lymphopaenia, and thrombocytopaenia. Blood glucose concentration on admission was 20.1 mmol/L with glycated haemoglobin (HbA1c) 12.4% (112 mmol/mol) and associated glycosuria. From day 17 of observation, the patient developed asymptomatic morning fasting hypoglycaemia, more frequently observed in the further course of treatment (glycaemic nadir 1.5 mmol/L). The concentration of endogenous insulin, measured after the reduction of insulin doses, when the patient required only low doses of prandial insulin, exceeded 300 μIU/mL. Fasting C-peptide concentration was within the normal range and increased adequately in glucagon stimulation test (0 min: 2.87 ng/mL, 6 min: 4.56 ng/mL). Plasma adiponectin concentration, assayed as previously described, was 13.8 mg/L (4). Insulin autoantibodies, glutamic acid decarboxylase autoantibodies and insulinoma-associated-2 autoantibodies were negative. Immunoprecipitation assay revealed anti-insulin receptor antibodies in two separate blood samples, confirming TBIR (Fig. 3). Hyperinsulinaemic euglycaemic clamping was performed in accordance with the protocol by DeFronzo *et al*. (5). The rate of whole-body glucose uptake (M value) was calculated as the mean glucose infusion rate during the last 40 min of the clamp, corrected for the glucose space and normalised for fat-free mass (FFM). The clamp confirmed severe insulin resistance with an M value of 2.1 mg/kgFFM/min (the mean M value obtained in a cohort of 60 healthy normal-weight men was 7.1 ± 2.4 mg/kgFFM/min (6)).

**Table 1:** Laboratory findings on admission.

Laboratory tests	Normal values	Results
HbA1c (mmol/mol)	26.8–44.3	112
Fasting glucose (mmol/L)	3.9–5.5	20.0
Fasting C-peptide (ng/mL)	0.78–5.19	2.87
C-peptide in glucagon test (6 min) (ng/mL)		4.56
Urine glucose	Negative	1000 mg/dL
Urine protein	Negative	25 mg/dL
Urine ketones	Negative	Negative
C-reactive protein (mg/L)	0.0–10.0	1.9
White blood cells (×10^3^/μL)	4.0–10.0	2.84
Red blood cells (×10^6^/μL)	4.5–6.0	4.09
Haemoglobin (g/dL)	14.0–18.0	12.5
Platelets (×10^3^/μL)	130–350	115
Total protein (g/dL)	6.0–8.0	6.4
IgG4 (g/L)	0.08–1.40	1.14
Sodium (mmol/L)	136.0–145.0	134
Potassium (mmol/L)	3.5–5.1	4.52
Alanine aminotransferase (IU/L)	5.0–50.0	68
Aspartate aminotransferase (IU/L)	5.0–50.0	36
Triglycerides (mg/dL)	<150	79
Total cholesterol (mg/dL)	<190	109
LDL-cholesterol (mg/dL)	<115	61
HDL-cholesterol (mg/dL)	>45	34
Insulin autoantibodies (U/mL)	<0.4	0.4
Anti-GAD antibodies (IU/mL)	<10	<5
Anti-IA-2 antibodies (IU/mL)	<10	<10
Anti-RNP/Sm antibodies	Negative	+++
Anti-Ro-52 antibodies	Negative	+++
Anti-MDA 5 antibodies	Negative	+
Anti-Ku antibodies	Negative	+
Anti-nuclear antibodies	<1:100	1:6400
Anti-neutrophil cytoplasmic antibodies	Negative	Negative
Anti-citrullinated peptides antibodies	Negative	Negative
C3 complement (mg/L)	970.0–1576.0	430
C4 complement (mg/L)	162.0–445.0	64
Anti-tTg (U/mL)	<0.3	0.2
Anti-dsDNA antibodies	Negative	+
Coombs direct test	Negative	Positive
Thyroid peroxidase antibodies (IU/mL)	0.0–5.61	1.26
Thyroglobulin antibodies (IU/mL)	0.0–4.11	2.61

GAD, glutamic acid decarboxylase; IA-2, insulinoma-associated protein-2; MDA-5, melanoma differentiation-associated gene 5; RNP, ribonucleoprotein; tTg, tissue transglutaminase.

Due to clinical features of connective tissue disease, a wider autoimmune screen was undertaken. High titres of antinuclear antibodies and anti-ribonucleoprotein antibodies were found, with positive Ro52 and anti-dsDNA antibodies. Direct Coombs test was positive, and serum complement concentrations were decreased. Collectively this led us to the diagnosis of mixed connective tissue disease. CT of the lungs and the abdomen, MRI of the brain, pulmonary function tests, and histological assessment of bone marrow, parotid glands and lymph nodes failed to demonstrate neoplasia.

## Treatment

**Figure 4:**
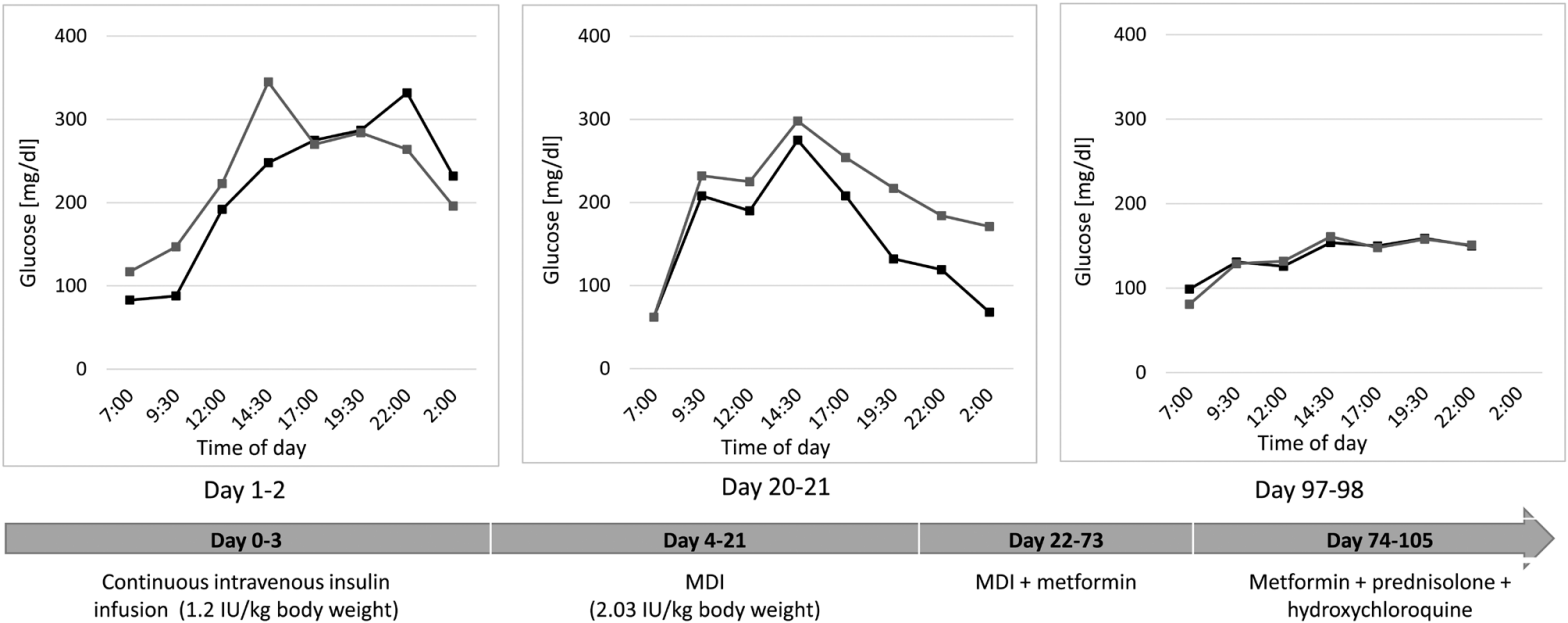
Daytime glycaemic profiles during the course of treatment. The blood glucose concentrations of 2 consecutive days are shown for appropriate intervention. MDI, multiple daily injections.

The treatment course is presented in Fig. 4. The patient was initially treated with i.v. insulin infusion, followed by intensive insulin therapy. This regimen did not improve blood glucose concentration despite daily insulin doses from 60 IU/24 h to 110 IU/24 h (>2 IU/kg of body weight). Metformin (850 mg three times a day) allowed gradual reduction of insulin doses (from 110 IU/24 h to 40 IU/24 h in 10 days). Given the coexistence of TBIR and mixed connective tissue disease, a cycle of pulsed methylprednisolone therapy was administered (250 mg/day for 3 days), followed by oral prednisone in decreasing doses for 1 month. Simultaneously, hydroxychloroquine (400 mg/day) was started and euglycaemia was maintained, which allowed withdrawal of insulin and reduction of metformin dose to 3 × 500 mg. Due to fasting morning hypoglycaemic episodes, the morning dose of metformin was stopped.

## Outcome and follow-up

**Figure 5:**
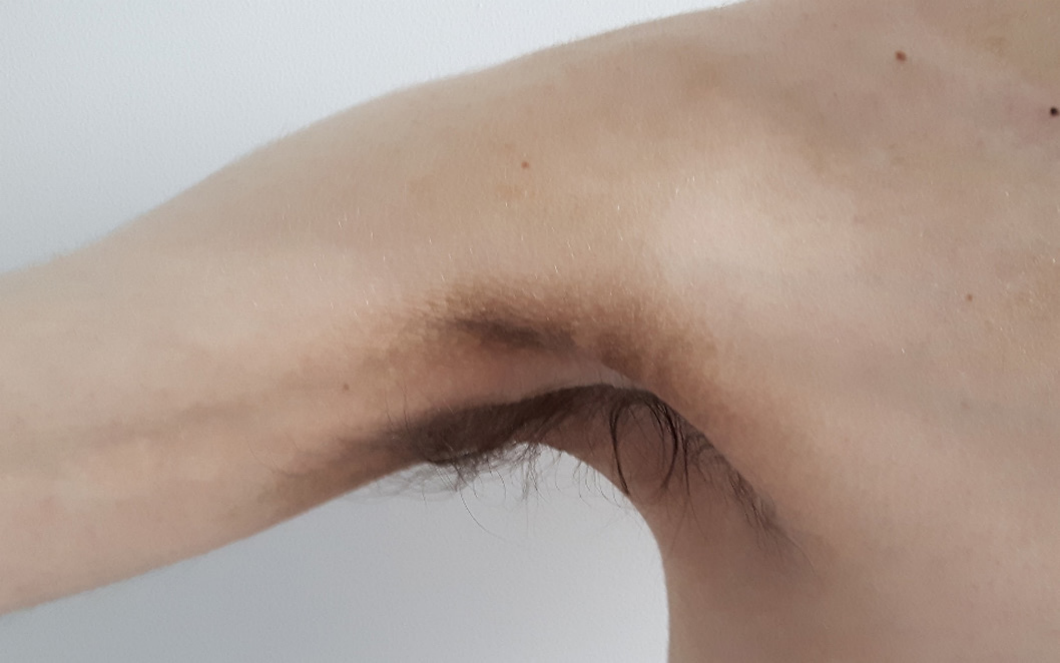
The amelioration of acanthosis nigricans after 6 months of treatment with glucocorticosteroids, hydroxychloroquine and metformin.

**Figure 6:**
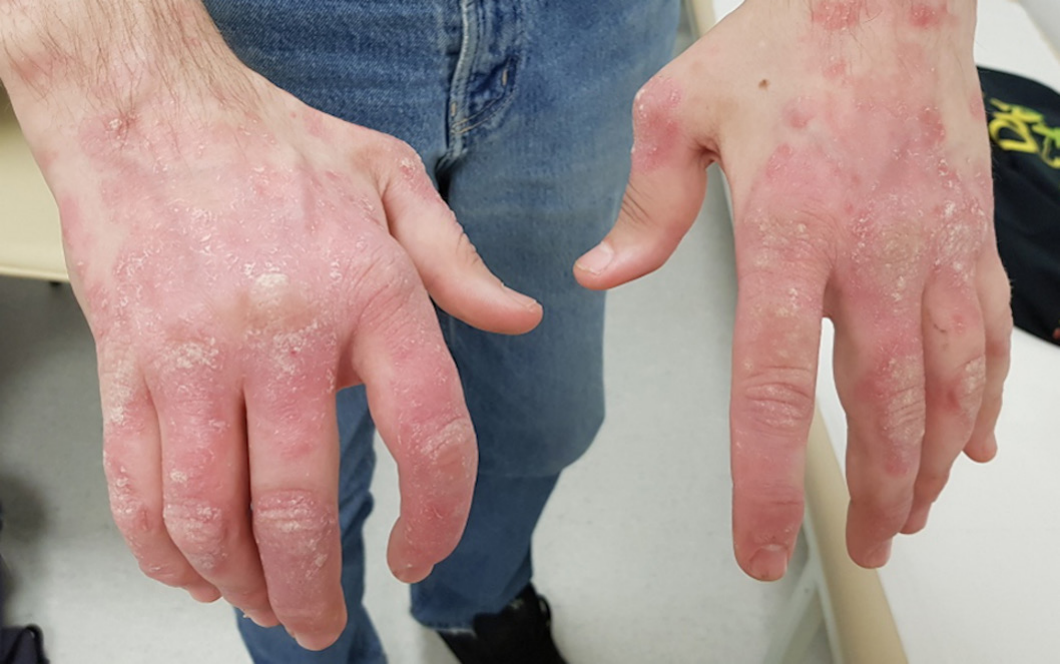
The extent of psoriatic lesions occurring during follow-up.

Six months after the initiation of treatment, weight had increased to 59.8 kg (BMI 18.5 kg/m^2^). Acanthosis nigricans and sclerodactyly also improved but did not remit entirely (Fig. 5). HbA1c decreased to 7.3% (56 mmol/mol) with only rare episodes of fasting hypoglycaemia. Fasting insulin concentration remained elevated at 126 µU/mL. The daily dose of metformin was reduced to 1000 mg/day in two doses. Thirty-three months later, inflammation of the elbow and interphalangeal joints of both hands developed with the intensification of psoriatic lesions (Fig. 6). Methotrexate treatment was initiated as an adjunct to hydroxychloroquine. During this therapy, weight gain increased further (BMI 19.7 kg/m^2^), with the disappearance of acanthosis nigricans, significant improvement of arthritis and reduction of psoriatic lesions. Fasting glucose concentration was 4.3 mmol/L.

## Discussion

Type B insulin resistance is rare. The largest longitudinal cohort of 24 patients has been reported by researchers from the United States National Institutes of Health (NIH) (1). It occurs mainly in the fourth to sixth decade of life, usually against a background of a rheumatological illness, most commonly connective tissue disease, lupus erythematosus, or as a paraneoplastic manifestation (1, 2, 7). We report a younger male Caucasian patient with typical symptoms of TBIR in association with mixed connective tissue disease and psoriasis. We assessed insulin sensitivity by hyperinsulinaemic euglycaemic clamp to confirm the severity of insulin resistance.

There is no pathognomonic clinical feature of TBIR, although rapid onset of extreme insulin resistance with widespread acanthosis nigricans, severe hyperglycaemia with attendant catabolic state and weight loss are characteristic. Type B insulin resistance syndrome is caused by AIRAs, which, based on rodent and cellular studies, act as agonists at the insulin receptor when the titre is low and as antagonists due to receptor downregulation when at high titre (8). This means that metabolic consequences of AIRAs are variable, ranging from extreme insulin resistance and hyperglycaemia in the first phase of the disease to recurrent fasting hypoglycaemia during early remission. Klubo-Gwiezdzinska *et al.* confirmed that all studied patients with TBIR experienced hypoglycaemic episodes when AIRA titre decreased, which allowed to reduce insulin doses (9). Initial presentation with hypoglycaemia alone is rare and was observed in only 13% of patients in the NIH cohort (1). Our patient started to develop fasting morning hypoglycaemia shortly after diabetes diagnosis. Despite reduction of insulin doses and eventually withdrawal of exogenous insulin, these episodes recurred. Thus, the suggestion of prolonged degradation of insulin–insulin receptor complexes or the coexistence of different populations of antibodies might be the possible pathomechanism (1).

The biochemical triad of markedly elevated fasting insulin concentration, hyperadiponectinaemia and low/normal fasting triglyceride concentrations was discussed as a ‘‘working" clinical definition of TBIR (10). Adiponectin level >7 mg/L in subjects with symptoms of severe insulin resistance had a 97% positive predictive value for defects of insulin receptor function (11). Our patient had high fasting insulin level, low triglycerides concentration and increased adiponectin concentration, which confirmed earlier findings.

Treatment of TBIR is challenging. The majority of reported cases of TBIR are associated with other autoimmune diseases, most often systemic lupus erythematosus or other connective tissue disease. To control hyperglycaemia at admission, the dose of insulin required intravenously in our patient was lower than presented in other cases, but was markedly higher than in "common" type 2 diabetes (7). Descriptions of use of metformin, sulphonylureas and thiazolidinediones in therapy of hyperglycaemia in TBIR have demonstrated variable efficacy (12, 13). In our patient, metformin allowed significant decrease in the dose of insulin.

The therapy requires intensive monitoring for side effects of immunosuppressive drugs and insulin titration due to glycaemic variability in the course of the disease (9). The NIH have recently proposed a treatment regimen consisting of rituximab, monthly high-dose glucocorticoids and cyclophosphamide, which has proved effective in an uncontrolled case series and allowed for discontinuation of insulin therapy in the studied patients (9). Combination immunosuppressive therapy, described in a prospective cohort study, followed by maintenance therapy with azathioprine, succeeded in reversing diabetes, signs of insulin resistance, and hyperandrogenism in women and was relatively safe (9). Unfortunately, the availability of rituximab in Poland is restricted in connective tissue disorders other than rheumatoid arthritis due to its high price. Other reported approaches to treatment have included use of prednisolone with hydroxychloroquine and azathioprine, plasmapheresis or i.v. immunoglobulin (3, 14, 15). In this case, corticosteroids and metformin permitted withdrawal of insulin and ameliorated insulin resistance symptoms. In several reported TBIR cases, remission has occurred spontaneously. In a cohort described by Arioglu *et al.*, spontaneous remission was observed in 33% of patients (1). Time to remission in this group ranged between 11 and 48 months and was similar to patients treated with immunosuppressive agents. Mortality rates in both groups were also comparable. However, in the case of coexisting disorders, such as connective tissue diseases, immunosuppressive treatment may be necessary to ameliorate their symptoms. In the presented case, the occurrence of psoriatic arthritis with connective tissue disease in 3 years of follow-up caused changes to the treatment regimen guided by a rheumatologist. Methotrexate with hydroxychloroquine helped reduce the severity of autoimmunological symptoms with concurrent decrease in the concentration of fasting insulin and reduction of hypoglycaemia episodes.

In conclusion, the presented case illustrates TBIR coexisting with other autoimmune conditions. Beyond the clinical signs of insulin resistance, hyperinsulinaemic euglycaemic clamp provided standardised biochemical confirmation. Targeted individualised therapy with a combination of metformin, hydroxychloroquine and methotrexate proved effective.

## Declaration of interest

The authors declare that there is no conflict of interest that could be perceived as prejudicing the impartiality of the research reported.

## Funding

Robert K Semple received funding from the Wellcome Trust (210752/Z/18/Z).

## Patient consent

Written informed consent to publish these findings was obtained from the patient.

## Author contribution statement

Agnieszka Łebkowska and Anna Krentowska were involved in diagnostic and therapeutic process and writing the article. Agnieszka Adamska, Danuta Lipińska, Beata Piasecka, and Maria Górska contributed to the diagnostic and therapeutic process. Otylia Kowal-Bielecka was involved in the diagnostic and therapeutic process and rheumatological consultations. Robert Semple was involved in the assessment of anti-insulin receptor antibodies and writing of the article. Irina Kowalska contributed to the diagnostic and therapeutic process, writing of the article, and final approval of the version to be submitted.

## References

[1] Arioglu E Andewelt A Diabo C Bell M Taylor SI Gorden P Clinical course of the syndrome of autoantibodies to the insulin receptor (type B insulin resistance): a 28-year perspective. Medicine 2002 81 87–100. (10.1097/00005792-200203000-00001) 11889410

[2] Braund WJ Naylor BA Williamson DH Buley ID Clark A Chapel HM Turner RC Autoimmunity to insulin receptor and hypoglycaemia in patient with Hodgkin’s disease. Lancet 1987 1 237–240. (10.1016/s0140-6736(87)90063-8) 2880067

[3] Kang SM Jin HY Lee KA Park JH Baek HS Park TS Type B insulin-resistance syndrome presenting as autoimmune hypoglycemia, associated with systemic lupus erythematosus and interstitial lung disease. Korean Journal of Internal Medicine 2013 28 98–102. (10.3904/kjim.2013.28.1.98) 23346003 PMC3543968

[4] Semple RK Soos MA Luan J Mitchell CS Wilson JC Gurnell M Cochran EK Gorden P Chatterjee VK Wareham NJ, Elevated plasma adiponectin in humans with genetically defective insulin receptors. Journal of Clinical Endocrinology and Metabolism 2006 91 3219–3223. (10.1210/jc.2006-0166) 16705075

[5] DeFronzo RA Tobin JD Andres R Glucose clamp technique: a method for quantifying insulin secretion and resistance. American Journal of Physiology 1979 237 E214–E223. (10.1152/ajpendo.1979.237.3.E214) 382871

[6] Stefanowicz M Nikolajuk A Matulewicz N Karczewska-Kupczewska M Adipose tissue, but not skeletal muscle, sirtuin 1 expression is decreased in obesity and related to insulin sensitivity. Endocrine 2018 60 263–271. (10.1007/s12020-018-1544-1) 29417372 PMC5893655

[7] Malek R Chong AY Lupsa BC Lungu AO Cochran EK Soos MA Semple RK Balow JE Gorden P Treatment of type B insulin resistance: a novel approach to reduce insulin receptor autoantibodies. Journal of Clinical Endocrinology and Metabolism 2010 95 3641–3647. (10.1210/jc.2010-0167) 20484479 PMC2913034

[8] Dons RF Havlik R Taylor SI Baird KL Chernick SS Gorden P Clinical disorders associated with autoantibodies to the insulin receptor. Simulation by passive transfer of immunoglobulins to rats. Journal of Clinical Investigation 1983 72 1072–1080. (10.1172/JCI111032) 6350362 PMC1129275

[9] Klubo-Gwiezdzinska J Lange M Cochran E Semple RK Gewert C Brown RJ Gorden P Combined immunosuppressive therapy induces remission in patients with severe Type B insulin resistance: a prospective cohort study. Diabetes Care 2018 41 2353–2360. (10.2337/dc18-0884) 30201849 PMC6196834

[10] Semple RK Halberg NH Burling K Soos MA Schraw T Luan J Cochran EK Dunger DB Wareham NJ Scherer PE, Paradoxical elevation of high-molecular weight adiponectin in acquired extreme insulin resistance due to insulin receptor antibodies. Diabetes 2007 56 1712–1717. (10.2337/db06-1665) 17325257 PMC2253187

[11] Semple RK Cochran EK Soos MA Burling KA Savage DB Gorden P O’Rahilly S Plasma adiponectin as a marker of insulin receptor dysfunction: clinical utility in severe insulin resistance. Diabetes Care 2008 31 977–979. (10.2337/dc07-2194) 18299442

[12] Fareau GG Maldonado M Oral E Balasubramanyam A Regression of acanthosis nigricans correlates with disappearance of anti-insulin receptor autoantibodies and achievement of euglycemia in type B insulin resistance syndrome. Metabolism: Clinical and Experimental 2007 56 670–675. (10.1016/j.metabol.2006.12.016) 17445543

[13] Gehi A Webb A Nolte M Davis J Treatment of systemic lupus erythematosus-associated type B insulin resistance syndrome with cyclophosphamide and mycophenolate mofetil. Arthritis and Rheumatism 2003 48 1067–1070. (10.1002/art.10879) 12687550

[14] Yang H Zhao J Li Y Lv F Zhang S Li Y Successful treatment of type B insulin resistance with mixed connective tissue disease by pulse glucocorticoids and cyclophosphamide. Journal of Diabetes Investigation 2017 8 626–628. (10.1111/jdi.12619) 28084020 PMC5497044

[15] Zhang S Wang G Wang J Type B insulin resistance syndrome induced by systemic lupus erythematosus and successfully treated with intravenous immunoglobulin: case report and systematic review. Clinical Rheumatology 2013 32 181–188. (10.1007/s10067-012-2098-x) 23053690

